# The Influence of Essential Oil Compounds on Antibacterial Activity of Mupirocin-Susceptible and Induced Low-Level Mupirocin-Resistant MRSA Strains

**DOI:** 10.3390/molecules24173105

**Published:** 2019-08-27

**Authors:** Paweł Kwiatkowski, Agata Pruss, Bartosz Wojciuk, Barbara Dołęgowska, Anna Wajs-Bonikowska, Monika Sienkiewicz, Monika Mężyńska, Łukasz Łopusiewicz

**Affiliations:** 1 Department of Diagnostic Immunology; Chair of Microbiology, Immunology and Laboratory Medicine; Pomeranian Medical University in Szczecin; 72 Powstancow Wielkopolskich Avenue, 70-111 Szczecin, Poland; 2 Department of Laboratory Medicine; Chair of Microbiology, Immunology and Laboratory Medicine; Pomeranian Medical University in Szczecin; 72 Powstancow Wielkopolskich Avenue, 70-111 Szczecin, Poland; 3 Institute of General Food Chemistry, Faculty of Biotechnology and Food Sciences; Lodz University of Technology; Stefanowskiego 4/10, 90-924 Lodz, Poland; 4 Department of Allergology and Respiratory Rehabilitation; Medical University of Lodz; Zeligowskiego 7/9, 90-752 Lodz, Poland; 5 Center of Bioimmobilisation and Innovative Packaging Materials; Faculty of Food Sciences and Fisheries; West Pomeranian University of Technology Szczecin; Janickiego 35, 71-270 Szczecin, Poland

**Keywords:** MRSA, mupirocin-susceptible, low-level mupirocin-resistant, essential oil compounds, synergistic activity, FTIR, Raman spectroscopy

## Abstract

Because of the bacterial drug resistance development, it is reasonable to investigate chemical compounds capable of preventing the spread of resistance to mupirocin (MUP), commonly used in staphylococcal eradication. The objective of the study was to verify the influence of essential oil compounds (EOCs) on the antibacterial activity of MUP against mupirocin-susceptible (MupS) and induced low-level mupirocin-resistant (MupRL) methicillin-resistant *Staphylococcus aureus* (MRSA) strains. The following parameters were examined: MRSA^MupS^ and MRSA^MupRL^ susceptibility to EOCs (1,8-cineole, eugenol, carvacrol, linalool, (-)-menthone, linalyl acetate, and *trans*-anethole), the bacterial cell size distribution, and chemical composition by the use of Fourier Transform Infrared Spectroscopy (FTIR) and Raman spectroscopies. The MRSA^MupS^ and MRSA^MupRL^ strains were susceptible to all tested EOCs. 1,8-cineole and (-)-menthone showed synergistic activity against MRSA^MupS^ in combination with mupirocin, whereas 1,8-cineole exhibited synergistic activity against MRSA^MupRL^ as well. In-depth analysis showed that both MRSA^MupS^ and MRSA^MupRL^ displayed similar distributions of the bacterial cell size. The FTIR and Raman spectra of the MRSA^MupS^ and MRSA^MupRL^ strains showed differences in some regions. New bands in the MRSA^MupRL^ Raman spectrum were observed. It was concluded that the use of 1,8-cineole in combination with mupirocin can increase the mupirocin activity against the MRSA^MupS^ and MRSA^MupRL^ strains.

## 1. Introduction

*Staphylococcus aureus* carriage is common in human populations. Different body areas may be colonized, but these bacteria are usually found in the nose vestibule. Approximately 20%–30% of healthy adults are persistent carriers, about 50% are transient carriers, and 20% have never been colonized by *S. aureus* [1]. Colonization with *S. aureus* is a major risk factor for staphylococcal infections. It is estimated that approximately 80% of staphylococcal sepsis is of an endogenous origin [2]. Nasal carriage, hair follicle infection (furuncle), or ongoing bone inflammation may appear as the source of sepsis. Staphylococcal infections may also result from direct or indirect contact with a carrier (exogenous infection). 

In order to eliminate the *S. aureus* carriage, as well as reduce the number of endogenous infections, topical mupirocin (an antibiotic produced by *Pseudomonas fluorescens*) ointment is applied to the nasal mucosa [3]. Decolonization is effective for patients who have completed the protocol, the duration of which typically ranges between 5 and 14 days [4]. The mechanism of mupirocin antibacterial activity is the inhibition of isoleucyl-tRNA synthetase (encoding by the *ileS* gene located on the chromosome) and associated bacterial protein synthesis blocking. There are two types of mupirocin resistance: low-level (MupRL, with a Minimum Inhibitory Concentration (MIC) between 8 and 256 mg/L) and high-level (MupRH, with a MIC of ≥512 mg/L). The MIC of mupirocin against susceptible strains is ≤4 mg/L [5]. MupRL is associated with *ileS* gene mutation. In turn, MupRH in *S. aureus* is mediated by a plasmid-encoded *ileS2* gene, which encodes an alternate isoleucyl-tRNA synthetase, with a very low affinity towards mupirocin [6].

The effective eradication of *S. aureus* from the nasal vestibule reduces the amount of bacteria colonizing the skin surface. This prevents staphylococcal post-operative wound infections and, at the same time, reduces the amount of bacteria transferred to the hands of medical personnel [7,8]. At present, screening for *S. aureus,* in particular, methicillin-resistant *S. aureus* (MRSA), carriage and the eradication of these strains in the host is ordered in patients before cardiac surgery and orthopedic procedures. In addition, it is strongly recommended to perform a full-body bath with the addition of disinfectant (e.g., chlorhexidine, triclosan, or povidone-iodine) before relevant surgical procedures. This is in order to reduce the number of infections caused by this particular organism.

In the era of increasing resistance to antibiotics, it becomes a new challenge to the scientific community to search for compounds that reduce the virulence of *S. aureus* strains and possibly make their eradication easier. Essential oil compounds (EOCs) appear to be good candidates for field testing to determine the effectiveness of antibacterial activity against many bacterial species, both susceptible and resistant to antibiotics. Due to their characteristic taste and aroma, EOCs are widely used in perfume and flavoring industries. In addition, EOCs are also being applied in cosmetic as well as medicine industries [9]. The existing studies have shown that EOCs have got many antimicrobial, antioxidant, anti-inflammatory, cancer chemoprotective, allelopathic, repellent, and insecticidal activities [10].

The aim of the study was to investigate the influence of EOCs on the antibacterial activity of mupirocin against mupirocin-susceptible and induced low-level mupirocin-resistant MRSA strains.

## 2. Results

### 2.1. The Antimicrobial Activity of Mupirocin against S. aureus ATCC 43300

It was found that mupirocin showed antibacterial activity against the reference strain. The obtained MIC (MIC_100_) value was 0.24 ± 0.00 mg/L. The MIC_50_ of mupirocin was calculated proportionally to the MIC_100_ value.

### 2.2. Emergence of Mutations under MIC_50_ of Mupirocin Pressure

After seven days of exposure to the MIC_50_ (0.12 mg/L) of mupirocin in Mueller–Hinton broth (MHB), it was noted that the reference strain showed an increase in its MIC, but remained in the susceptible range (3.91 mg/L). It was also found that the control strain in MHB without mupirocin had the same MIC value as described previously (0.24 mg/L). After this period, the new MIC_50_ (1.95 mg/L) of mupirocin was calculated proportionally to the old MIC value as described above. This strain was exposed to the new MIC_50_ of mupirocin for seven additional days. It was again observed that exposure to the new MIC_50_ of mupirocin increased in the MIC during the second exposure and remained in the low-level resistance range (31.25 mg/L). After this time, the MIC of the control strain was still 0.24 mg/L. 

The strain susceptible to mupirocin was designated as MRSA^MupS^ (MIC = 0.24 mg/L), and with induced low-level mupirocin-resistance as MRSA^MupRL^ (MIC = 31.25 mg/L).

### 2.3. The Antimicrobial Effects of EOCs against MRSA^MupS^ and MRSA^MupRL^

It was proven that both MRSA^MupS^ and MRSA^MupRL^ strains were susceptible to all tested EOCs. The highest inhibiting activity was observed for carvacrol (MIC = 0.48 ± 0.00–0.95 ± 0.00 mg/mL). In contrast, the lowest antibacterial activity of *trans*-anethole (MIC = 494.0 ± 0.0 mg/mL) was revealed. The results of the MICs of EOCs against the MRSA^MupS^ and MRSA^MupRL^ strains are summarized in Table 1.

### 2.4. Combination of EOCs and Mupirocin against MRSA^MupS^ and MRSA^MupRL^

The study indicated that 1,8-cineole showed synergistic activity in combination with mupirocin against both MRSA^MupS^ and MRSA^MupRL^ strains. Moreover, synergistic action was also demonstrated by (-)-menthone against the MRSA^MupS^ strain, in contrast to the MRSA^MupRL^ (antagonistic effect). The following EOCs (eugenol, carvacrol, and *trans*-anethole) exhibited the additive effect in combination with mupirocin against the MRSA^MupRL^ strain, whereas the same EOCs (eugenol, carvacrol) presented antagonistic and indifferent effects against the MRSA^MupS^ strain. The results for the checkerboard assay against the MRSA^MupS^ and MRSA^MupRL^ strains are listed in Table 1.

### 2.5. A Comparison of Cell Size between MRSA^MupS^ and MRSA^MupRL^

**Figure 1:**
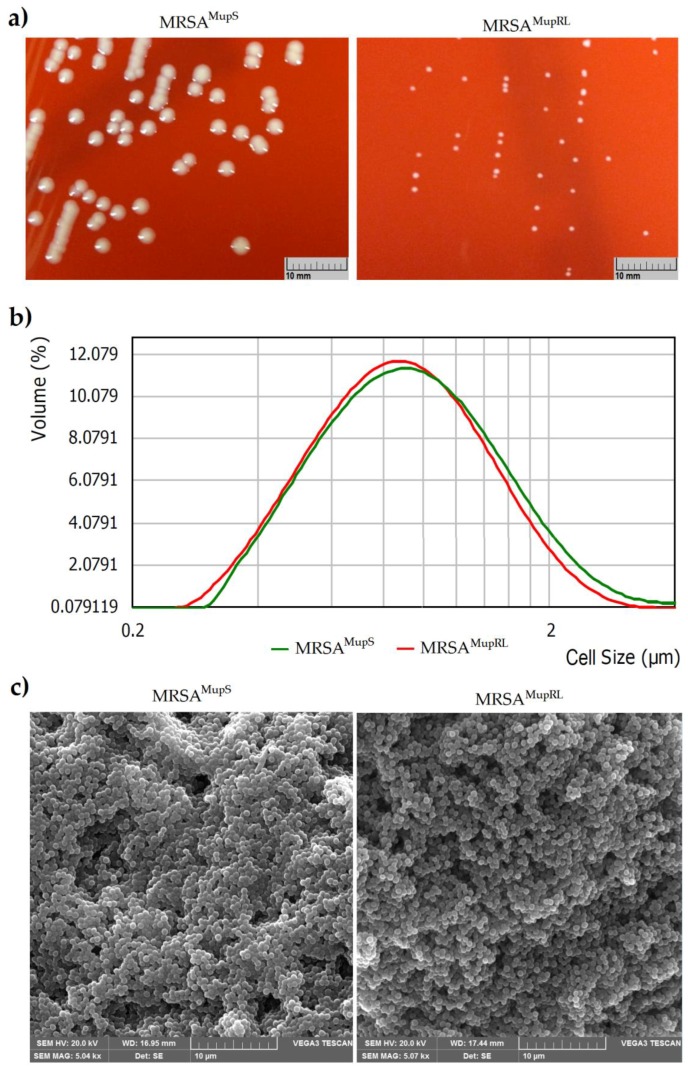
Comparison of the sizes of growing MRSA^MupS^ and MRSA^MupRL^ colonies on Columbia agar with 5% sheep blood (**a**), and cells using Mastersizer 2000 (**b**), and SEM (**c**).

It was found that the size of the bacterial colonies differed between the analyzed strains (susceptible to mupirocin and with induced low-level mupirocin-resistance) cultivated on Columbia agar with 5% sheep blood (Figure 1A). For this reason, it was assumed that the difference in colony size between these strains could be connected with the size of cells. However, an in-depth analysis showed that both MRSA^MupS^ and MRSA^MupRL^ presented a similar distribution of the bacterial cell size, respectively 0.838 ± 0.116 µm for the susceptible and 0.806 ± 0.132 µm for the intermediate to mupirocin strain (Figure 1B). It was also observed using Scanning Electron Microscope (SEM) (Figure 1C).

### 2.6. FTIR Analysis

**Figure 2:**
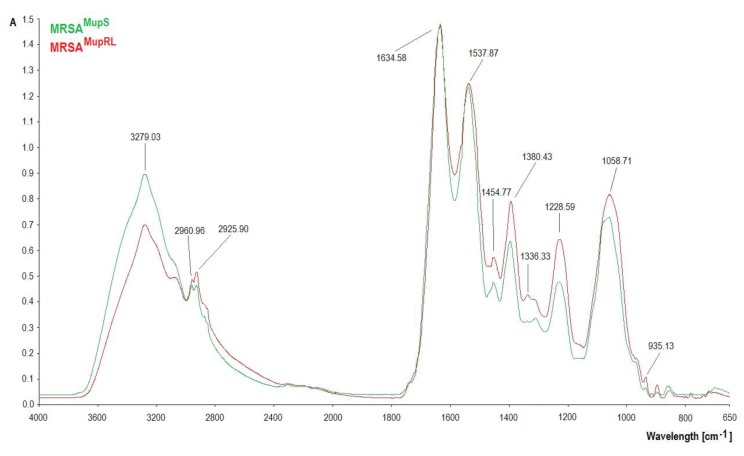
FTIR spectra of MRSA^MupS^ and MRSA^MupRL^ strains.

The FTIR spectra of the MRSA^MupS^ and MRSA^MupRL^ strains are shown in Figure 2. The differences were observed particularly in the complex spectral region at 1700–1200 cm^−1^ and at 1200–900 cm^−1^. At 3279.03 cm^−1^ the absorbance value of MRSA^MupS^ was higher than MRSA^MupRL^. A noticeable growth of absorbance at bands 2960.96 cm^−1^ and 2925.90 cm^−1^ was observed. No changes were observed at 1634.58 cm^−1^. In the amide II region, MRSA^MupRL^ absorbance increased at 1454.77 cm^−1^ and 1380.43 cm^−1^. At 1228.59 cm^−1^ and at 1058.71 cm^−1^, there was a decrease in absorbance in the MRSA^MupS^ sample. It was also observed that absorbance increased in MRSA^MupRL^ in the narrow region centered at 935.13 cm^−1^.

### 2.7. Raman Analysis

**Figure 3:**
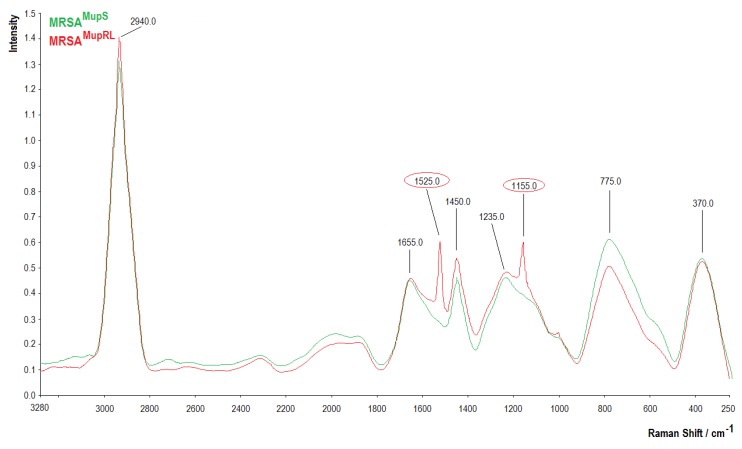
Raman spectra of MRSA^MupS^ and MRSA^MupRL^ strains.

The Raman spectra of the MRSA^MupS^ and MRSA^MupRL^ strains are presented in Figure 3. Between 600 and 900 cm^−1^, centered around 775.0 cm^−1^, a higher band in the MRSA^MupS^ sample was noted. A new band at 1155.0 cm^−1^ in the MRSA^MupRL^ sample was observed. Higher bands at 1235.0 cm^−1^ and 1450.0 cm^−1^ were noticed in the MRSA^MupRL^ sample in comparison to the MRSA^MupS^ sample. In the region between 1500 and 1800 cm^−1^, a new band at 1525.0 cm^−1^ was noted in the MRSA^MupRL^ sample, whereas in both samples, a similar intensity of band at 1655.0 cm^−1^ was observed. In the region between 2800 and 3100 cm^−1^, an intense signal was noted for the MRSA^MupRL^ sample.

## 3. Discussion

Scientific research with modern methodology has confirmed the valuable properties of many medicinal plants known for ages in medicine. The development of accompanying sciences has led to research on plant-originated compounds, providing reliable scientific data. There has been a lot of research that has proven multidirectional activity and the potential of plant-derived compounds. Essential oils—secondary metabolites of plants—have been of increasing interest in recent years. According to Nazzaro et al., essential oils are classified as “Generally Recognised as Safe” (GRAS) by the Food and Drugs Administration (FDA). These are not harmful and are more widely accepted by consumers [11]. These represent anticancer and anti-diabetic activities and are recommended for cardiovascular diseases including atherosclerosis and thrombosis [12]. In this research, the main constituents of the following essential oils were studied: *Eucalyptus globulus* Labill. (1,8-cineole, content not less than 70%), *Syzygium aromaticum* (L.) Merr. & Perry, *Eugenia caryophyllata* Thunb. (eugenol, required from 75% to 88%), *Lavandula angustifolia* Mill. (required: linalool from 20% to 45% and linalyl acetate from 25% to 46%), *Mentha × piperita* L. (menthone required from 14% to 32%), *Foeniculum vulgare* Mill. (*trans*-anethole required from 60% to 80%), and *Thymus vulgaris* L. (carvacrol, required 40%) [13]. Because of their high antimicrobial activity, they bring hope to stop the growth of resistance among pathogenic microorganisms to synthetic drugs. Combinatorial studies, which involve essential oil blends, showed their synergistic action against multidrug-resistant pathogens like MRSA, *Escherichia coli*, producing extended spectrum beta-lactamases, and also against yeasts or dermatophytes [14,15,16].

The prevalence of high and low-level mupirocin resistance affects not only *S. aureus*, but also coagulase-negative staphylococci—a reservoir of conjugative plasmids of mupirocin resistance frequently responsible for serious infections, especially in immunocompromised hosts and patients treated with implants and catheters [17]. Due to the easy spread of staphylococci in the hospital environment and their ability to survive on non-living surfaces, it is reasonable to look for compounds that will prevent the spread of resistance to mupirocin used for staphylococcal eradication in patients and hospital staff. Data collected by Khoshnood et al. showed that the emergence of mupirocin resistance is currently increasing mostly among MRSA isolates [18].

In this study the influence of the following essential oil dominant constituents (1,8-cineole, eugenol, carvacrol, linalool, (-)-menthone, linalyl acetate, and *trans*-anethole) on the antibacterial activity of mupirocin against mupirocin-susceptible and induced low-level mupirocin-resistant MRSA strains was investigated. This investigation was further deepened by the spectral analysis of the tested strains believing that changes in the structures of outer bacterial cell membranes may affect the penetration of essential oil components and, in consequence, the mupirocin activity.

This research showed the differences in FTIR and Raman spectra between MRSA^MupS^ and MRSA^MupRL^ strains. The differences in FTIR spectra between MRSA^MupS^ and MRSA^MupRL^ strains were observed particularly in the complex spectral region at 1700–1200 cm^−1^ where many types of vibrations were present (CH_2_ bending, COO- stretching, PO_2_^−^ stretching) and at 1200–900 cm^−1^, corresponding to the stretching and bending vibrations of C-O-C and C-OH groups and the C-O ring vibrations of the polysaccharide region attributed to carbohydrates in the bacterial cell wall [19]. At 3279.03 cm^−1^, the absorbance value of MRSA^MupS^ was higher than MRSA^MupRL^, suggesting more hydroxyl groups in the MRSA^MupS^ strain. A noticeable growth of absorbance at bands 2960.96 cm^−1^ and 2925.90 cm^−1^, respectively, was attributed to CH_2_ and CH_3_ groups. No changes were observed at 1634.58 cm^−1^ (amide I), attributed to proteins. In the amide II region, MRSA^MupRL^ absorbance increased at 1454.77 cm^−1^ and 1380.43 cm^−1^, the bands that are attributed to the asymmetric bending of methyl groups in proteins and lipids, and stretching the C-N vibrations of cytosine-guanine pairs. At 1228.59 cm^−1^, imputed to the asymmetric stretching of the phosphodiester backbone, there was a decrease in absorbance in MRSA^MupS^ [20]. At 1058.71 cm^−1^, which was attributed to stretching OH coupled with bending the CO of the polysaccharides capsule and peptidoglycan and stretching the CO of polysaccharides, a decline in MRSA^MupS^ absorbance was noted [21]. The absorbance increase in MRSA^MupRL^ in the narrow region centered at 935.13 cm^−1^, assignable to C-O-C and C-O-P symmetric stretching in cell wall oligosaccharides and polysaccharides, was also noticed [20].

The differences in the Raman spectra between the MRSA^MupS^ and MRSA^MupRL^ strains were observed between 600 and 900 cm^−1^, centered around 775 cm^−1^, a noticeably higher band in the MRSA^MupS^ sample—related to DNA and RNA, attributed to adenine containing species, to a mode of adenine ring breathing in DNA and DNA phosphodiester stretching, out-of-plane ring breathing modes of tyrosine, and to the C-O-C stretching vibration of 1,4 glycosidic link in carbohydrates [22,23]. The region between 900 and 1500 cm^−1^ was characterized by a series of emission bands from amides, lipids, and carbohydrates. A new band at 1155 cm^−1^ in MRSA^MupRL^ was related to C-O-C and =C-C= antisymmetric stretching in aliphatic esters and the glycosidic link of carbohydrates. The band 1235 cm^−1^ higher in the MRSA^MupRL^ sample was related to C-N stretching in the amide III region [23]. At 1450 cm^−1^, the higher intensity in the MRSA^MupRL^ sample can be attributed to CH_2_ vibrations from membrane lipids and polysaccharides such as poly-N-acetylglucosamine. The region between 1500 and 1800 cm^−1^ contained one α-helix-related band (C=O and -C=C stretch) at 1525 cm^−1^ in the MRSA^MupRL^ sample. In both samples, a similar intensity of the β-sheet-related C=O stretching emission of amide I (proteins) and in lipids at 1655 cm^−1^ was noted [23,24]. In the region between 2800 and 3100 cm^−1^, a more intense signal was noticed for the MRSA^MupRL^ sample, attributed to the symmetric and antisymmetric stretching of CH_2_ and CH_3_ in lipids, fatty acids, proteins, and carbohydrates [23].

The mupirocin molecule consists of 9-hydroxy nonanoic acid (a short fatty acid side-chain) and monic acid (a C polyketide-derived substructure), which are linked by an unsaturated ester linkage [18]. Presenting a negative charge, mupirocin enters the bacterial cells by passive diffusion in an energy-independent manner [25,26]. The pivotal factors which determine the resistance to mupirocin are potentially the following: lower affinity to the antibiotic, higher affinity to the substrate, overproduction of the synthetase enzyme, decreased permeability of the bacterial membranes, or a combination of these [18,25]. Based on the spectral analysis of the MRSA^MupS^ and MRSA^MupRL^ strains, it can be concluded that resistance to mupirocin may affect the chemical composition of bacterial cells. Those changes, particularly related to the polysaccharides and peptidoglycan of the capsule, as well as cell wall oligosaccharides and polysaccharides, may result in different electrostatic interactions with anionic antibiotic molecules, leading to its lower penetration into the MRSA^MupRL^ strain.

The current research confirmed the thesis of the various activities of essential oils and their components in combination with antibacterial compounds against susceptible and resistant bacteria of the same species. Our previous study showed that *trans*-anethole appeared efficient in increasing susceptibility to mupirocin and decreasing biofilm formation in mupirocin-resistant *S. aureus* strains [27]. It has also been observed in this study. *Trans*-Anethole presented indifferent and addictive effects against the MRSA^MupS^ and MRSA^MupRL^ strains, respectively. Moreover, another study showed the good antibacterial activity of terpenoids against the MRSA^MupS^ and MRSA^MupRL^ strains and also confirmed the special properties of 1,8-cineole as a recognized permeation enhancer, described by Hendry et al. in his research on MRSA [28]. Kifer et al. did not notice the difference in the activity of EOCs (menthol and 1,8-cineole) towards mupirocin in planktonic MRSA strains, but the mixture of mupirocin and thymol had an inconclusive effect. They also observed that 1,8-cineole exerted a potentiated biofilm-eliminating effect [29]. Other research also showed the increased antimicrobial activity of 1,8-cineole in combination with chlorhexidine gluconate (CHG) against MRSA strains [30]. According to Xu et al., *E. globulus* essential oil contains mainly 1,8-cineole. This is recommended as a good remediation against respiratory infectious diseases, also for children, and is a component of many medicinal mixtures. The authors observed that a dose range of 0–64.15 mg/kg/day did not cause even slight organ damage in mice [31]. The current study has shown that the most effective combination of 1,8-cineole and mupirocin decreased the MIC of 1,8-cineole from 307.00 ± 132.93 to 57.56 ± 0.00 mg/mL and from 57.56 ± 0.00 to 14.39 ± 0.00 mg/mL, respectively, for the MRSA^MupS^ and MRSA^MupRL^ strains. Yadav et al. showed the very good antibacterial activity of eugenol, alone or in combination with carvacrol against methicillin-susceptible *S. aureus* (MSSA) and MRSA clinical strain biofilms. They found that eugenol significantly decreased the expression of biofilm- and enterotoxin-related genes, and decreased bacterial colonization in the middle ear as well. The authors recommended that eugenol be administered alone or in combination with carvacrol in *S. aureus* biofilm-related infections. Moreover, eugenol is approved as a safe food preservative by the European Union and FDA. It has been extensively applied in stomatology as well [32].

Despite the evident differences in subcellular structures between the two tested strains, no particular differences were observed regarding their physical properties apart from colony size. Moreover, these revealed similar susceptibility to the tested combinations of mupirocin with particular EOCs. Trombetta et al. proposed a mechanism of the antimicrobial effect of three monoterpenes (menthol, thymol, and linalyl acetate) [33]. These compounds may be responsible for the membrane permeability and leakage of intracellular materials. Furthermore, the authors supposed that these effects could be dependent on the lipid composition and net surface charge of microbial membranes. The study showed that the differences between FTIR and Raman spectra provided by both strains indicate cellular wall variability, which may possibly contribute both to mupirocin and EOCs’ susceptibility. It is clearly visible in the mupirocin-(-)-menthone combination, which showed synergy and an antagonistic effect against MRSA^MupS^ and MRSA^MupRL^, respectively. 

Still more precise fingerprinting methods are needed to identify the therapeutic targets for new drugs or new formulas of known drugs, e.g., combinations between the conventional antibiotics and EOCs. Targeting molecular patterns is appearing outstandingly in emerging studies on drugs like mupirocin, which is not decisive for survival in individual cases but represents a significant tool in preventive procedures in terms of the spread of drug resistance.

## 4. Materials and Methods

### 4.1. Bacterial Strain and Growth Conditions

The *S. aureus* ATCC 43300 (MRSA) was used in this study. The reference strain was cultivated for 18 h at 37 °C on Columbia agar with 5% sheep blood (bioMérieux, Warsaw, Poland) and then a single colony was inoculated into Mueller–Hinton broth (MHB, Sigma-Aldrich, Darmstadt, Germany) and cultured for 18 h at 37 °C.

### 4.2. Chemicals

Concentrations of mupirocin (≥92% purity, obtained through HPLC, Sigma-Aldrich, Germany) from 250 to 0.12 mg/L were prepared by dissolving the drug in dimethyl sulfoxide (DMSO, Loba Chemie, Mumbai, India) (2%, *v*/*v*) and diluting by MHB. Concentrations of EOCs—1,8-cineole (99% purity, Ernesto Ventos S.A., Barcelona, Spain), eugenol (≥98% purity, Ernesto Ventos S.A., Spain), carvacrol (99% purity, Sigma-Aldrich, Germany), linalool (≥96% purity, Ernesto Ventos S.A., Spain), (-)-menthone (96% purity, Ernesto Ventos S.A., Spain), linalyl acetate (≥97% purity, Ernesto Ventos S.A., Spain), and *trans*-anethole (99% purity, Sigma-Aldrich, Germany) from 500 to 0.12 µL/mL—were prepared by dissolving compounds in Tween 80 (Sigma-Aldrich, Germany) (1%, *v*/*v*) and diluting by MHB.

### 4.3. Determination of Minimum Inhibitory Concentration (MIC) of Mupirocin against S. aureus ATCC 43300

The MIC of mupirocin was determined by the broth microdilution method in a 96-well microplate according to the Clinical and Laboratory Standards Institute [34]. Each well contained 50 µL of the appropriate mupirocin concentration and 50 µL of the *S. aureus* ATCC 43300 suspension at a final concentration of 10^6^ CFU/mL. Staphylococcal suspension was performed for 18-hour cultures using MHB. The MIC was estimated as described earlier [35]. To exclude an inhibitory effect of 2% (*v*/*v*) DMSO on the reference strain growth, the control assays with MHB and MHB supplemented with DMSO were performed. All tests were carried out in triplicate.

### 4.4. Induction of Mutations

The induction of mutations in the *S. aureus* ATCC 43300 strain was performed according to Lee et al. [36] with minor modification. *S. aureus* ATCC 43300 was incubated in MHB (control) and MHB supplemented with the MIC_50_ of mupirocin according to the original MIC of the reference strain. After 18 h of incubation at 37 °C, the suspensions were adjusted to a 0.5 McFarland scale and diluted again (1:100) in fresh MHB. This assay was repeated on a daily basis for seven days to reflect the duration of in vivo exposure to mupirocin in eradication protocols from the nose vestibule (about 14 days). Then the reference strain underwent MIC determinations by the broth microdilution method described above. After seven days, the strain was subjected to another seven-day mupirocin exposure cycle, with the drug concentration adjusted according to the new MIC. The obtained MIC was estimated by the broth microdilution method as described previously (in Section 4.3). At this stage, the strain susceptible to mupirocin (control) was designated as MRSA^MupS^, and with induced low-level mupirocin resistance as MRSA^MupRL^.

### 4.5. Determination of MIC of EOCs against MRSA^MupS^ and MRSA^MupRL^ Strains

The MIC values of the following EOCs (1,8-cineole, eugenol, carvacrol, linalool, (-)-menthone, linalyl acetate, and *trans*-anethole) against the MRSA^MupS^ and MRSA^MupRL^ strains were determined by the broth microdilution method according to Clinical and Laboratory Standards Institute (CLSI) [34] with a minor modification: a final concentration of sterile 1% (*v*/*v*) Tween 80 was incorporated into MHB to enhance the EOCs’ solubility. All tests were performed as described above (in Section 4.3). To exclude an inhibitory effect of 1% (*v*/*v*) Tween 80 on the MRSA^MupS^ and MRSA^MupRL^ strains’ growth, the control assays with MHB and MHB supplemented with 1% (*v*/*v*) Tween 80 were performed. Using the known concentrations of EOCs, the final result was expressed in mg/mL.

### 4.6. Checkerboard Method

The checkerboard method was performed against the MRSA^MupS^ and MRSA^MupRL^ strains as previously described [37]. Two-fold dilutions (250–0.12 mg/L for mupirocin and 500–0.12 µL/mL for each EOC) were performed. Each well contained the following components: 25 μL of the appropriate concentration of EOC, 25 μL of the appropriate mupirocin concentration, and 50 µL of bacterial suspension containing the final concentration of 10^6^ CFU/mL in each well. The plates were incubated at 37 °C for 18 h. All assessments were performed in duplicates. Using the known densities of the EOCs, the final result was expressed in mg/mL. The MIC of both mupirocin and the EOCs combined or without combination were determined using resazurin as described in the previous study [35]. The combined effects of mupirocin and the EOCs were calculated and expressed in terms of a fractional inhibitory concentration index (FICI) using the following formula:

$$\mathrm{FIC}\;=\;\frac{\mathrm{MIC}\;\mathrm{of}\;\mathrm{EOC}\;\mathrm{or}\;\mathrm{mupirocin}\;\mathrm{in}\;\mathrm{combination}\;}{\mathrm{MIC}\;\mathrm{of}\;\mathrm{EOC}\;\mathrm{or}\;\mathrm{mupirocin}\;\mathrm{alone}}$$

FICI = FIC of EOC + FIC of mupirocin

Results were interpreted as follows: synergy (FICI < 0.5), addition (0.5 ≤ FICI ≤ 1.0), indifference (1.1 < FICI ≤ 4.0), or antagonism (FICI > 4.0) [37].

### 4.7. Bacterial Cell Size Distribution

The bacterial cell size distribution was analyzed by the use of the particle size analyzer Mastersizer 2000 (Malvern Panalytical Ltd., Malvern, United Kingdom). The bacteria (MRSA^MupS^ and MRSA^MupRL^) were cultivated for 18 h at 37 °C on Columbia agar with 5% sheep blood. After incubation, grown colonies were harvested with a loop and suspended in sterile phosphate-buffered saline (PBS, pH 7.4). Cells were centrifuged (5000 rpm for 5 min) and washed three times with PBS. After this, bacteria were suspended initially in PBS with 0.01% sodium dodecyl sulfate solution (SDS, Sigma-Aldrich, Germany) to reach concentration 7 in a McFarland scale (approx. 2.1 × 10^9^ CFU/mL), then vigorously vortexed for 1 min. The bacterial suspensions were then dispersed in 800 mL (stirrer speed—2000 rpm) of distilled water at room temperature (20 °C) to reach obscurance in range 5.0%. Each sample was measured in triplicate.

### 4.8. A Determination of Functional Groups in Staphylococcal Cells by the Use of Fourier Transform Infrared (FTIR) and Raman Spectroscopy

In order to confirm the presence of particular chemical moieties in MRSA^MupS^ as well as MRSA^MupRL^ cells, FTIR and Raman spectroscopy analyses were carried out. After the cultivation of these strains for 18 h at 37 °C on Columbia agar with 5% sheep blood, the cells were washed three times by 5 mL of PBS, centrifuged at 5000 for 5 min, and dried for 24 h at 37 °C. The FTIR spectra of bacterial cells dry samples were obtained at room temperature by attenuated total reflection with a FTIR spectrometer (Perkin Elmer Spectrophotometer 100, Waltham, MA, USA). The samples (100 mg) were then scanned at a range between 650 cm^−1^ and 4000 cm^−1^ (64 scans and 1 cm^−1^ resolution). The obtained spectra were normalized, baseline corrected, and analyzed using SPECTRUM software (v10, Perkin Elmer Spectrophotometer, Waltham, MA, USA).

To obtain Raman spectra, the samples were analyzed using a Raman spectrometer (RamanStation 400F, Perkin Elmer, USA) with point and shot capability using an excitation laser source at 785 nm (to avoid fluorescence excitation), 100 micron spot size, 4 shots, and 8 s exposition time. The obtained spectra were normalized, baseline corrected, and analyzed using SPECTRUM software (v10, Perkin Elmer, Waltham, MA, USA).

### 4.9. Scanning Electron Microscopy (SEM)

The SEM observations were carried out according to Xu et al. [38] with slight modification. The bacteria (MRSA^MupS^ and MRSA^MupRL^) were incubated for 18 h at 37 °C on Columbia agar with 5% sheep blood. Then, the bacterial colonies were collected directly from the media surface and washed three times with 0.1 M PBS and centrifuged (1500 rpm for 10 min). Glutaraldehyde (2.5%, *v*/*v*) (Chempur, Piekary Slaskie, Poland) was used to fix the bacterial cells for 4 h at 4 °C. The samples were firstly dehydrated in a gradient ranging ethanol (30%–100%, *v*/*v*) (Chempur, Poland) and then the ethanol was gradually replaced with the same ranging tert-butanol (Chempur, Poland) at room temperature. The samples were dried with vacuum freeze-drying equipment for 8 h. Finally, bacterial cells were coated with gold for 90 s and a scanning electron microscope Vega 3 LMU (Tescan, Brno, Czech Republic) was used to observe.

### 4.10. Statistical Analysis

All data were expressed as mean ± standard deviation (SD).

## 5. Conclusions

The EOCs analyzed in this study increased the mupirocin susceptibility of both mupirocin-susceptible and induced low-level mupirocin-resistant MRSA strains. This refers mostly to 1,8-cineole. Also, both staphylococcal strains revealed differences in spectroscopic profiles. These point to a cellular wall structure, however, these do not significantly affect either physical properties or mupirocin and EOC susceptibility. More precise high throughput technologies are needed to target the checkpoints crucial for staphylococcal mupirocin resistance and its control.

## Figures and Tables

**Table 1:** Fractional inhibitory concentration (FIC) and FIC indices (FICI) of mupirocin-essential oil compound (EOC) pairs against mupirocin-susceptible (MRSA^MupS^) and induced low-level mupirocin-resistant (MRSA^MupRL^) strains.

Strains	Mupirocin-EOCs	MIC_O_	MIC_C_	FIC	FICI	Type of Interaction
MRSA^MupS^	Mupirocin-1,8-cineole					
	Mupirocin (mg/L)	0.24 ± 0.00	0.06 ± 0.00	0.25	0.44	synergy
	1,8-cineole (mg/mL)	307.00 ± 132.93	57.56 ± 0.00	0.19		
	Mupirocin-eugenol					
	Mupirocin (mg/L)	0.24 ± 0.00	0.24 ± 0.00	1.0	5.0	antagonism
	Eugenol (mg/mL)	2.08 ± 0.00	8.34 ± 0.00	4.0		
	Mupirocin-carvacrol					
	Mupirocin (mg/L)	0.24 ± 0.00	0.24 ± 0.00	1.0	5.0	antagonism
	Carvacrol (mg/mL)	0.95 ± 0.00	3.81 ± 0.00	4.0		
	Mupirocin-linalool					
	Mupirocin (mg/L)	0.24 ± 0.00	0.24 ± 0.00	1.0	1.5	indifference
	Linalool (mg/mL)	6.8 ± 1.0	3.4 ± 0.0	0.5		
	Mupirocin-(-)-menthone					
	Mupirocin (mg/L)	0.24 ± 0.00	0.06 ± 0.00	0.25	0.38	synergy
	(-)-Menthone (mg/mL)	27.91 ± 0.00	3.49 ± 0.00	0.13		
	Mupirocin-linalyl acetate					
	Mupirocin (mg/L)	0.24 ± 0.00	0.12 ± 0.00	0.5	1.5	indifference
	Linalyl acetate (mg/mL)	450.5 ± 0.0	450.5 ± 0.0	1.0		
	Mupirocin-*trans*-anethole					
	Mupirocin (mg/L)	0.24 ± 0.00	0.24 ± 0.00	1.0	2.0	indifference
	*trans*-Anethole (mg/mL)	494.0 ± 0.0	494.0 ± 0.0	1.0		
MRSA^MupRL^	Mupirocin-1,8-cineole					
	Mupirocin (mg/L)	31.25 ± 0.00	0.98 ± 0.00	0.03	0.28	synergy
	1,8-cineole (mg/mL)	57.56 ± 0.00	14.39 ± 0.00	0.25		
	Mupirocin-eugenol					
	Mupirocin (mg/L)	31.25 ± 0.00	15.63 ± 0.00	0.5	1.0	addition
	Eugenol (mg/mL)	8.34 ± 0.00	4.17 ± 0.00	0.5		
	Mupirocin-carvacrol					
	Mupirocin (mg/L)	31.25 ± 0.00	15.63 ± 0.00	0.50	0.75	addition
	Carvacrol (mg/mL)	0.48 ± 0.00	0.12 ± 0.00	0.25		
	Mupirocin-linalool					
	Mupirocin (mg/L)	31.25 ± 0.00	62.5 ± 0.0	2.0	11.6	antagonism
	Linalool (mg/mL)	2.83 ± 0.98	27.19 ± 0.00	9.6		
	Mupirocin-(-)-menthone					
	Mupirocin (mg/L)	31.25 ± 0.00	62.5 ± 0.0	2.0	18.0	antagonism
	(-)-Menthone (mg/mL)	6.98 ± 0.00	111.63 ± 0.00	16.0		
	Mupirocin-linalyl acetate					
	Mupirocin (mg/L)	31.25 ± 0.00	31.25 ± 0.00	1.0	2.0	indifference
	Linalyl acetate (mg/mL)	450.5 ± 0.0	450.5 ± 0.0	1.0		
	Mupirocin-*trans*-anethole					
	Mupirocin (mg/L)	31.25 ± 0.00	7.81 ± 0.00	0.25	0.74	addition
	*trans*-Anethole (mg/mL)	494.0 ± 0.0	247.0 ± 0.0	0.49		

Values are expressed as mean ± standard deviation. MICo, MIC of EOC or mupirocin; MICc, MIC of EOC/mupirocin combination. FIC index = FIC of EOC + FIC of mupirocin. FICI < 0.5, synergy; 0.5 ≤ FICI ≤ 1.0, addition; 1.1 < FICI ≤ 4.0, indifference; FICI > 4.0, antagonism. Using the known concentrations of EOCs, the final result was expressed in mg/mL.
